# Predicting neurodevelopmental outcomes in Australian First Nations infants: The transdiagnostic utility of early screening tools

**DOI:** 10.1111/dmcn.70003

**Published:** 2025-09-25

**Authors:** Carly Luke, Katherine A. Benfer, Leeann Mick‐Ramsamy, Robert S. Ware, Margot Bosanquet, Natasha Reid, Arend F. Bos, Roslyn N. Boyd, Queensland State‐wide LEAP Clinical Group, Shaneen Leishman, Anya Gordon, Hailey Williams, Chloe Taifalos, Maria Smith, Donna Dewis, Ellena Oakes, Lynda McNamara, Megan Kentish

**Affiliations:** ^1^ Queensland Cerebral Palsy and Rehabilitation Research Centre, Child Health Research Centre, Faculty of Health, Medicine and Behavioural Sciences The University of Queensland Brisbane QLD Australia; ^2^ Queensland Paediatric Rehabilitation Service Children's Health Queensland Hospital and Health Service Brisbane QLD Australia; ^3^ Griffith Biostatistics Unit Griffith University Brisbane QLD Australia; ^4^ Department of Health and Wellbeing Townsville Hospital and Health Service District (THHS) Townsville QLD Australia; ^5^ Child Health Research Centre, Faculty of Health, Medicine and Behavioural Sciences The University of Queensland Brisbane QLD Australia; ^6^ Beatrix Children's Hospital, Division of Neonatology University of Groningen Groningen the Netherlands

## Abstract

**Aim:**

To determine the predictive relationship between evidence‐based screening tools and neurodevelopmental outcomes in Australian First Nations infants.

**Method:**

This prospective cohort study invited First Nations families to participate in a culturally adapted early developmental screening programme. A total of 156 infants (55.1% male, mean gestational age = 33.6 weeks, SD = 4.6) were screened using the Prechtl's General Movements Assessment, with optimality scoring using the Motor Optimality Score‐Revised (MOS‐R) at 3 to 5 months and the Hammersmith Infant Neurological Examination (HINE) at 4 to 9 months. Participants completed ‘baby movement (BM) checks’ at two time points (BM1, 3–5 months corrected age; BM2, 4–9 months corrected age), with final movement and learning checks at 12 months corrected age. At 12 months corrected age, standardized motor, cognitive, and communication assessments, neurodisability‐specific symptomology, or a diagnosis made by a paediatrician classified infants as developing typically (‘on track’) or (1) with a high chance of or confirmed cerebral palsy (CP) or (2) non‐CP neurodevelopmental delay (NDD), including autism and fetal alcohol spectrum disorder (FASD). Predictive relationships were investigated using logistic regression and diagnostic statistics.

**Results:**

At 12 months, 127 of 147 (86%) eligible infants (*n* = 9 withdrawn or deceased) were classified as ‘on track’ (*n* = 55, 43%), NDD (*n* = 59, 47%), or CP (*n* = 13, 10%). MOS‐R (≥ 14 weeks) and the HINE distinguished infants as ‘on track’, CP, or NDD. [Correction added on 1 November 2025 after first online publication: In the preceding sentence, “MOS‐R (≥ 14 weeks). The HINE distinguished infants…” has been updated to “MOS‐R (≥ 14 weeks) and the HINE distinguished infants…”.] Each 1‐point decrease on both tools increased the odds of NDD (OR_MOS‐R_ = 1.40, 95% confidence interval [CI] = 1.00–1.96; OR_HINE_ = 1.12, 95% CI = 1.05–1.21) and CP (OR_MOS‐R_ = 1.47, 95% CI = 1.08–2.01; OR_HINE_ = 1.41, 95% CI = 1.21–1.65,). The MOS‐R (cut‐off of less than 23) and HINE (moderate to severely reduced) were best for identifying any NDD and CP (MOS‐R: sensitivity = 84%, specificity = 38%; HINE: sensitivity = 64%, specificity = 63%). Combined trajectories across both tools were the strongest predictors of CP (sensitivity = 73%, specificity = 96%), autism (sensitivity = 59%, specificity = 95%), and FASD (sensitivity = 89%, specificity = 93%).

**Interpretation:**

Evidence‐based screening tools demonstrate promising transdiagnostic prediction of ‘on‐track’ development and not only high chance of CP but also autism, FASD, and other NDDs.

## Graphical abstract

A culturally adapted developmental screening program (LEAP‐CP) improved First Nations families' engagement with neonatal follow‐up by almost 40%. The Motor Optimality Score‐Revised (MOS‐R), Hammersmith Infant Neurological Examination (HINE), and the Australian First Nations adaptation of the Ages and Stages Questionnaire (ASQ‐TRAK), distinguished infants developing ‘on track’ (typically) from those with neurodevelopmental concerns, including high chance of cerebral palsy, autism, and fetal alcohol spectrum disorder at 12 months corrected age. Combined trajectories across the MOS‐R and HINE provided the strongest early markers of developmental concerns, supporting timely referral and culturally responsive access to early support and intervention services.

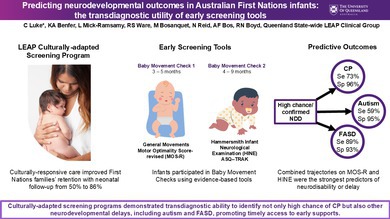

Plain language summary: https://onlinelibrary.wiley.com/doi/10.1111/dmcn.70072

**This original article is commented on by Yasumitsu‐Lovell on pages** 305–306 **of this issue.**

**Abbreviations**
ASQ‐TRAK: Ages and Stages Questionnaire‐Talking about Raising Aboriginal Kids
Bayley‐III: Bayley Scales of Infant and Toddler Development, Third Edition
FASD: fetal alcohol spectrum disorder
GMA: Prechtl's General Movements Assessment
HINE: Hammersmith Infant Neurological Examination
LEAP‐CP: Learning through Everyday Activities with Parents for infants with CP
MOS‐R: Motor Optimality Score‐Revised
NDD: neurodevelopmental delay
PDMS‐2: Peabody Developmental Motor Scales, Second Edition
PPV: positive predictive value
SACS‐R: Social Attention and Communication Surveillance‐Revised

**What this paper adds**

- The Motor Optimality Score‐Revised (MOS‐R) and Hammersmith Infant Neurological Examination (HINE) distinguished between infant 12‐month neurodevelopmental outcomes.
- Evidence‐based tools demonstrate promising transdiagnostic prediction of high chance of autism and fetal alcohol spectrum disorder, not just cerebral palsy.
- Combined trajectories across the MOS‐R and HINE were the strongest early biomarkers for neurodevelopmental outcomes.
- A culturally adapted screening programme improved Australian First Nations families' retention with neonatal follow‐up by 35%.

A strong start to life promotes long‐term health and well‐being, enhancing a child's opportunity to thrive. Infants born early, with low birthweight, or requiring support in hospital after birth experience an increased chance of neurodevelopmental delay (NDD) or disability, including cerebral palsy (CP), autism, and fetal alcohol spectrum disorder (FASD). Enhancing early childhood development is a priority for First Nations communities;^1^, ^2^ however, families experience barriers to accessing equitable, culturally safe, and responsive health care.^3^, ^4^ In Australia, they demonstrate low engagement (approximately 50%) with neonatal screening programmes.^5^ Developmental screening aims to distinguish typically developing infants (‘on track’), from those requiring extra support to move, learn, and grow strong.

A transdiagnostic approach to developmental screening acknowledges that many NDDs share common early markers and comorbidities across multiple domains.^6^, ^7^ Although conditions such as CP, autism, and FASD have distinct aetiologies and clinical presentations, they often exhibit overlapping features in early neurodevelopmental profiles.^6^, ^8^, ^9^ Differences in movement quality, atypical motor development, and cognitive functioning are common early indicators across these conditions.^6^, ^10^, ^11^ To be effective, early screening programmes should incorporate tools that are predictive, valid, feasible, culturally safe, and capable of identifying an infant's risk status for a range of neurodevelopmental differences, supporting timely referral to intervention services, even before a specific diagnosis is established.^7^ There are few clinical biomarkers of non‐CP NDDs or neurodiverse outcomes, including autism and FASD, before 12 months corrected age, delaying timely access to early intervention. Evidence‐based tools such as the Prechtl's General Movements Assessment (GMA), the associated Motor Optimality Score‐Revised (MOS‐R), and the Hammersmith Infant Neurological Examination (HINE) demonstrate high predictive accuracy for the early detection of CP^12^, ^13^ and may provide key transdiagnostic indicators of non‐CP NDDs,^14^, ^15^ and motor and cognitive delays.^16^, ^17^, ^18^, ^19^

This research respectfully involved and engaged Australian First Nations families and communities, representing a diversity of Aboriginal and Torres Strait Islander peoples across Queensland, Australia. At the forefront of change is acknowledging and implementing culturally led health services, embedding First Nations peoples' priorities, world views, and ways of being, knowing, and doing, into clinical service delivery. Rethinking and adapting health service models requires consideration of the key structural and cultural barriers and enablers for engagement and improving care.^20^, ^21^ In a culturally adapted developmental screening programme for First Nations infants, we aimed to determine the transdiagnostic accuracy of early screening tools to predict neurodevelopmental outcomes at 12 months corrected age. We hypothesized that fidgety movements GMA would demonstrate the greatest predictive ability for a high chance of CP while detailed scoring on the MOS‐R and HINE would demonstrate the strongest predictive accuracy for later NDDs, differentiating infants developing ‘on track’ from those with a higher chance of CP or NDD, including autism and FASD.

## METHOD

This prospective cohort study invited First Nations families to participate in a culturally adapted early developmental screening programme^5^, ^22^ (Learning through Everyday Activities with Parents for infants with CP [LEAP‐CP]) as per protocol (no. ANZTR12619000969167).^5^ The study was reported in line with the STrengthening the Reporting of OBservational studies in Epidemiology (STROBE) guidelines, with the CONSolIDated critERia for strengthening the reporting of health research involving Indigenous Peoples (CONSIDER) statement^23^ guiding culturally responsive and ethical research practices involving First Nations peoples.

First Nations communities across participating Queensland sites were actively involved at every stage of the research programme. Key stakeholders, including Elders, First Nations health workers, researchers, and parents with lived experience, informed research priorities, contributed to study design, and guided culturally safe approaches to early screening, recruitment, consent, and communication. Ongoing consultation informed the development and delivery of the programme, ensuring that First Nations priorities and perspectives were embedded throughout.

Eligible participants were recruited by allied health clinicians and First Nations health workers before 9 months corrected age (birth years 2020–2022) from health services across three main sites, Wulgurukaba and Bindal country (Townsville), Gimuy, Walubara, Yidinji, and Yirrganydji country (Cairns), and Yugera and Turrbal country (Brisbane), in Queensland, Australia, to include a representative population of First Nations families across urban, regional, rural, and remote contexts. Infants with increased developmental vulnerability because of medical, birth, or post‐neonatal risk factors for NDDs and with one or both biological parents who self‐identified as Aboriginal or Torres Strait Islander were eligible. Infants were excluded if major congenital or chromosomal abnormalities were identified as part of routine medical care.^5^

Ethics approval for the LEAP‐CP study was obtained through the Far North Queensland Health Research Ethics Committee (HREC/2019/QCH/50533 [Sep ver 2]—1370), the Townsville Hospital and Health Service Human Research Ethics Committee (no. HREC/QTHS/56008), the University of Queensland Medical Research Ethics Committee (no. 2020000185/HREC/2019/QCH/50533), and the Children's Health Queensland Hospital and Health Service Human Research Ethics Committee (no. HREC/20/QCHQ/63906). Research governance approval was obtained from the Apunipima Cape York Health Council Research and Gurriny Yealamucka Health Services Aboriginal Corporation. Parents or caregivers provided written informed consent. Our culturally adapted programme aimed to promote First Nations families' engagement with, and access to, existing health services by designing a programme that used evidence‐based tools, co‐designed and encompassed within a First Nations framework (led by LMR), and embedded into current models of care. Both culturally specific (use of engaging language, co‐designed educational resources, building capacity of First Nations health workers and cultural engagement training for clinicians) and clinically specific (consistent and relationally focused workforce, flexibility in service delivery, use of culturally adapted tools) changes to current health service models were incorporated to create a new, culturally responsive model of care for early screening.^22^ Infants deemed to be at high risk of CP or NDD based on early screening outcomes were offered participation in the LEAP‐CP early support programme.^24^

Participants completed ‘baby movement (BM) checks’ at two time points (BM1, 3–5 months corrected age; BM2, 4–9 months corrected age), with final movement and learning checks at 12 months corrected age. At BM1, infants completed the GMA (two videos between 12 weeks and 16 weeks corrected age) at clinic appointments, home visits, or via the Baby Moves app.^25^ Advanced GMA‐trained assessors blinded to the infant's medical history assessed the GMA (fidgety movements and MOS‐R) and gained a consensus score. At BM2, a HINE and the culturally validated and adapted version of the Ages and Stages Questionnaire‐Talking about Raising Aboriginal Kids (ASQ‐TRAK) were administered. Infant demographic, perinatal, and cultural data (cultural identity, family connections to country, and languages spoken at home) were collected via interview with a First Nations health worker or local clinician. Data were entered into a Research Electronic Data Capture (REDCap) database (Vanderbilt University, Nashville, TN, USA).

The GMA included scoring of fidgety movements, classified as normal (continuous or intermittent) or aberrant (abnormal or absent/sporadic), and detailed optimality scoring on the MOS‐R. The MOS‐R assesses the infant motor repertoire using five subcategories: fidgety movements; observed movement patterns; age‐adequate motor repertoire; observed postural patterns; and movement character. It takes on average 9 minutes to score.^26^ Subcategories are scored as typical/optimal (score of 4), suboptimal/reduced (score of 2), or absent/atypical (score of 1), with fidgety movements more weighted (normal [score of 12], abnormal [score of 4], or absent [score of 1]). Subcategories were combined to obtain a total MOS‐R (range = 5–28), with scores further classified as optimal (≥ 25), mild (20–24), moderate (9–19), or severely reduced (≤8).^27^, ^28^

The HINE is a quick to administer neurological assessment (it takes approximately 15 minutes), validated to evaluate infant neuromotor outcomes from 2 to 24 months corrected age across five subcategories: cranial nerve function; posture; movements; tone; and reflexes and reactions.^29^ HINE global scores (maximum = 78) were compared to age‐specific optimality and cut‐off scores for CP or significant delay^16^, ^29^, ^30^, ^31^, ^32^ and classified as optimal (> 64, > 68, > 71, ≥ 73),^29^, ^30^ mild (< 10th centile; 58–64, 64–68, 69–71, 69–72), moderate (less than the significant delay cut‐off score; 57, 60–63, 63–68, 66–70),^16^, ^31^ or severely reduced (less than the CP cut‐off score; < 57, < 60, < 63, < 66)^32^ at 4 to 9 months and 12 months respectively (Figure S1). Differences in item responses between the left and right sides were recorded as asymmetries.

The ASQ‐TRAK is validated to screen First Nations children (aged 2 months–5 years 6 months) across five developmental domains: communication; gross motor; fine motor; personal‐social; and problem‐solving.^33^, ^34^ Domain‐specific scores (maximum = 60) were compared to the domain cut‐off scores to identify developing ‘on‐track’ infants or developmental differences.^35^

At 12 months corrected age, infants completed final movement and learning checks on the Bayley Scales of Infant and Toddler Development, Third Edition (Bayley‐III) (cognition and communication), the Peabody Developmental Motor Scales, Second Edition (PDMS‐2) (gross and fine motor), and the HINE (neurological) with local allied health clinicians. The likelihood of autism was screened using the Social Attention and Communication Surveillance‐Revised (SACS‐R).^36^ To account for the local clinicians not being blinded to the early assessment outcomes, 10% of the Bayley‐III, HINE, and PDMS‐2 were randomly selected and scored by assessors blinded to infant early outcomes, and medical and social history, to determine interrater reliability.

The Bayley‐III is a norm‐referenced measure of infant cognitive, language, motor, and social–emotional development.^37^ A composite score 85 or less (>1 SD below the mean) identifies the presence of language or cognitive delay. The PDMS‐2, a standardized, norm‐referenced measure, evaluates gross and fine motor development in children aged 0 to 6 years. Composite quotient scores of 85 or less (>1SD below the mean) identify the presence of gross, fine, or total motor delays. The SACS‐R is a highly accurate screening tool for identifying infants with a high likelihood of autism from 11 months corrected age.^36^ In 13 511 Australian children aged 11 to 24 months, the SACS‐R demonstrated 99% specificity and 62% sensitivity for predicting a later confirmed diagnosis of autism at 4 years,^36^ with the positive predictive value (PPV) increasing across age points—lowest at 12 months (74%) and higher at 18 (82%) and 24 (86%) months. Importantly, none of the infants assessed as having a high likelihood of autism were developing typically by age 4 years. This finding reinforces the clinical value of the SACS‐R in identifying infants who may benefit from timely access to early intervention services, even when diagnostic certainty is not yet established.

Neurodevelopmental status was classified via paediatrician diagnosis as confirmed or high chance of CP or other non‐CP NDDs (FASD, autism, global developmental delay, or mild‐to‐severely reduced scores on a motor [PDMS‐2] composite quotient score of 85 or less), communication or cognitive (Bayley‐III composite score of 85 or less) domains. For infants with outcomes on the ASQ‐TRAK only, NDD was defined as one or more domains below the cut‐off (severely reduced, >2SD below the mean) or two or more domains close to the cut‐off (mild‐to‐moderately reduced, 1‐2SD below the mean).^35^ Infants were classified at 12 months corrected age as either developing typically (‘on track’) or (1) high chance/confirmed CP or (2) other non‐CP NDDs (Figure S1).

In addition to identifying infants at high chance of NDD, we aimed to evaluate the predictive value of early screening tools for high chance/confirmed autism and FASD, and the prognostic indicators of functional severity and topography in CP. For the secondary analyses, infants with NDD‐specific features were further classified as high chance of autism based on the SACS‐R, or high chance of FASD based on (1) paediatrician diagnosis; (2) presence of FASD‐specific facial features; or (3) confirmed prenatal alcohol exposure with severe delay on three or more developmental domains.^38^ For infants with high‐chance/confirmed CP, functional severity was classified according to the Gross Motor Function Classification System (GMFCS) for the age band of 0 to 2 years.

### Statistical analysis

Participant perinatal and demographic characteristics are described using summary statistics. The association between continuous variables and neurodevelopmental outcomes were evaluated using the Kruskal–Wallis test, with Dunn's post‐hoc test, and the Mann–Whitney *U* test. The association between categorical variables and neurodevelopmental outcomes was evaluated using the Fisher's exact test. Regression modelling was used to determine the relationship between early screening assessments (fidgety movements, MOS‐R, HINE, ASQ‐TRAK) and infant outcomes at 12 months corrected age. For three‐level outcomes (developing ‘on track’ vs high‐chance/confirmed CP vs NDD), multinomial logistic regression models were used; for two‐level outcomes (developing ‘on track’ vs high‐chance/confirmed NDD/CP, for autism or for FASD) logistic regression models were used. Multivariable models were constructed to understand which screening tools demonstrated the greatest association with outcomes at each time point (baby movement check). Model 1 (BM1 [Multi_BM1]) included the MOS‐R < 14 weeks and > 14 weeks as covariates. Model 2 (BM2 [Multi_BM2]) included the HINE and ASQ‐TRAK as covariates. Model 3 (all screening tools, Multi_all) included all covariates from models 1 and 2. Diagnostic statistics, including sensitivity, specificity, positive and negative predictive values, and receiver operator characteristic curve analysis were calculated. Significance was set at *α* = 0.05 (two‐tailed). Each analysis used all available data. Missing data were not imputed. Statistical analysis was performed using Stata v18 (StataCorp, College Station, TX, USA).

## RESULTS

Of the 160 infants referred for screening, 156 (97.5%) (55.1% male, mean gestational age 33.6 weeks [SD = 4.6]) participated. Of these, 139 of 148 (94%) eligible infants completed the BM1 (fidgety movements, MOS‐R), 140 of 150 (93%) eligible infants completed the BM2 (HINE, ASQ‐TRAK), and 127 of 147 (86%) eligible infants (*n* = 9 withdrawn or deceased) completed the outcomes at 12 months corrected age (Figure S2). A sample size of 120 infants was calculated^5^ to provide sufficient power (*α* = 0.05, power = 0.80) to evaluate the diagnostic accuracy of early screening tools and detect statistically significant associations with neurodevelopmental outcomes at 12 months corrected age.

Participant characteristics, demographics, and participation in the baby movement checks are reported in Table 1. More infants completed the GMA at 14 weeks or more (T2; *n* = 118) compared to fewer than 14 weeks (T1; *n* = 94), with 82 (60%) capturing the GMA at both time points. Of the 127 infants with 12‐month outcomes, 101 (80%) completed the PDMS‐2 and Bayley‐III for the cognitive (*n* = 100, 79%) and communication (*n* = 99, 78%) domains. Older maternal age (*p* = 0.012), cultural and linguistic connection in the home (*p* = 0.047), and living with extended or community‐based caregivers (e.g. kinship care, *p* = 0.014), were positively associated with sustained engagement in developmental screening at 12 months (Table S1). Inter‐assessor reliability was excellent (intraclass correlation coefficient = 0.97–0.98) across all domains on the PDMS‐2, Bayley‐III, and HINE.

**TABLE 1:** Infant characteristics, early life risk factors, and demographics (*n* = 156).

	With 12‐month outcomes (*n* = 127)	Without 12‐month outcomes (*n* = 29)	Missing/not completed, *n* (%)
Eligible for neonatal follow‐up, *n* = 83	71 (55.9)	12 (41.4)	NA
Gestational age (weeks), range (23–42), mean (SD)	33.6 (4.6)	34.5 (4.2)	NA
VPT (< 32 weeks)	41 (32.3)	7 (24.1)	NA
Birthweight (g), mean (SD)	2165.2 (988.6)	2271.2 (945.7)	NA
VLBW (< 1500 g)	35 (27.6)	6 (20.7)	NA
IUGR	33 (26.0)	7 (24.1)	6 (4.0)
Sex (male)	72 (57.0)	14 (48.3)	NA
Cultural identity^a^			
Aboriginal	58 (45.7)	23 (79.3)	NA
Torres Strait Islander	11 (8.6)	2 (6.9)	NA
Both	58 (45.7)	4 (13.8)	NA
Multiple birth	35 (27.5)	4 (13.8)	NA
HIE	5 (3.9)	2 (6.9)	NA
Moderate to severe	3 (60.0)	0 (0)	1 (20.0)
Therapeutic cooling	3 (60.0)	1 (50.0)	NA
Seizures	10 (7.8)	3 (10.3)	NA
Bronchopulmonary dysplasia	23 (18.11)	4 (14.3)	1 (6.4)
Neonatal surgery	18 (14.1)	1 (3.4)	7 (4.5)
Post‐neonatal event	5 (3.9)	2 (6.9)	NA
Neuroimaging	75 (59.1)	13 (44.8)	NA
CUS	67 (89.3)	12 (92.3)	NA
MRI	23 (30.6)	6 (46.1)	NA
Both	15 (20.0)	5 (38.4)	NA
IVH	13 (10.2)	1 (3.4)	NA
PVL	2 (1.6)	1 (3.4)	NA
Stroke	0 (0)	0 (0)	NA
Other brain abnormalities (i.e. cortical malformation)	6 (4.7)	0 (0)	NA
Geographical location^a^			
Urban	14 (11.0)	0 (0)	NA
Regional	108 (85.0)	25 (86.2)	NA
Rural/remote	5 (3.9)	4 (13.8)	NA
BM1 (3–5 months), *n* = 138	113 (90.4)	25 (86.2)	NA
GMA < 14 weeks, *n* = 94	79 (62.2)	15 (51.7)	NA
Aberrant fidgety movements	12 (15.1)	3 (18.8)	NA
MOS‐R mean (SD)	20.3 (4.9)	19.2 (7.0)	NA
GMA ≥ 14 weeks, *n* = 117	97 (76.9)	20 (70.0)	NA
Aberrant fidgety movements	13 (13.4)	4 (13.8)	NA
MOS‐R, mean (SD)^a^	20.1 (4.8)	17.0 (6.4)	NA
BM2 (4–9 months), *n* = 140	120 (94.5)	18 (62.0)	NA
Global HINE score, mean (SD)	63.5 (10.0)	62.8 (13.9)	NA
Corrected age at HINE (months), mean (SD)	6.06 (1.3)	5.9 (1.3)	NA
Neurodevelopmental outcome at 12 months	127 (100.0)	0 (0)	29 (100.0)
'On track' (developing typically)	55 (43.3)	NA	NA
Neurodevelopmental delay	59 (46.5)	NA	NA
Cerebral palsy	13 (10.2)	NA	NA

Abbreviations: BM1, baby movement check 1; BM2, baby movement check 2; CP, cerebral palsy, CUS, cranial ultrasound; GMA, Prechtl's General Movements Assessment; HIE, hypoxic‐ischaemic encephalopathy; HINE, Hammersmith Infant Neurological Examination; IUGR, intrauterine growth restriction; IVH, intraventricular haemorrhage; MOS‐R, Motor Optimality Score‐Revised; MRI, magnetic resonance imaging; PVL, periventricular leukomalacia; VLBW, very low birthweight; VPT, very preterm.

Data are *n* (%) unless stated otherwise.

^a^ Significant difference (*p* < 0.05).

### Prediction of neurodevelopmental outcomes

The 12‐month outcomes of infants were classified as 55 (43%) infants developing ‘on track’, 59 (47%) infants with confirmed/high chance of NDD, and 13 (10%) infants with confirmed/high chance of CP (Table 1). Baby movement check results are presented in Table 2. MOS‐R scores at 14 weeks or longer significantly differed between outcome groups, with ‘on‐track’ infants demonstrating higher scores (mean [SD] = 22.1 [2.2]) than those at high chance of NDD (20.4 [3.7], *p* = 0.036) and CP (11.4 [6.2], *p* < 0.001). HINE global scores were significantly higher for infants ‘on track’ (68.3 [4.7]) than those at high chance of NDD (63.7 [7.5], *p* = 0.001) and CP (45.3 [13.1], *p* < 0.001), regardless of age at assessment. The age at which the HINE assessment was conducted did not differ significantly between outcome groups (*p* = 0.91).

**TABLE 2:** GMA (fidgety movements, MOS‐R), HINE, and ASQ‐TRAK according to outcome group (*n* = 127).

	All infants *n* = 127	'On‐track'' infants *n* = 55 (43.3)	NDD *n* = 59 (46.5)	Cerebral palsy *n* = 13 (10.2)	*p*
Eligible for neonatal follow‐up, *n* = 71	71 (55.9)	32 (58.2)^a^	22 (37.3)^a^	11 (84.6)^a^	0.008^b^
BM1, *n* = 113	113 (89.0)	47 (85.5)	55 (93.2)	11 (84.6)	NA
GMA <14 weeks, *n* = 79	79 (69.9)	30 (63.8)	42 (76.4)	7 (63.6)	NA
Fidgety movements					
Normal	67 (84.8)	28 (93.3)^a^, ^c^	37 (88.1)^a^, ^c^	2 (28.6)^a^	<0.001^b^
Abnormal	2 (2.5)	1 (3.2)	1 (2.4)	0 (0)	NA
Absent	10 (12.5)	1 (3.2)^a^, ^c^	4 (9.5)^a^, ^c^	5 (71.4)^a^	<0.001^b^
MOS‐R					
Total score, mean (SD)	20.1 (5.3)	21.6 (4.0)^a^, ^c^	20.8 (3.9)^a^, ^c^	11.7 (5.5)^a^	<0.001^d^
Median (IQR)	21 (20–23)	21 (21–24)	21 (21–23)	11 (6–18)	NA
GMA ≥ 14 weeks, *n* = 97	97 (85.8)	42 (89.4)	44 (80.0)	11 (100.0)	NA
Fidgety movements					
Normal	84 (86.6)	42 (100.0)^a^, ^c^	39 (88.6)^a^, ^c^	3 (27.3)^a^	<0.001^b^
Abnormal	2 (2.1)	0 (0)	2 (4.5)	0 (0)	NA
Absent	11 (11.3)	0 (0)^a^, ^c^	3 (6.9)^a^, ^c^	8 (72.7)^a^	<0.001^b^
MOS‐R					
Total score, mean (SD)	19.6 (5.2)	22.1 (2.2)^a^	20.4 (3.7)^a^	11.4 (6.2)^a^	<0.001^d^
Median (IQR)	21 (20–22)	21 (20–24)	21 (20–21.5)	9 (6–20)	NA
BM2, *n* = 120	120 (94.5)	51 (92.7)	58 (98.3)	13 (100.0)	NA
HINE, *n* = 118	118 (92.9)	49 (89.0)	56 (94.9)	13 (100.0)	NA
Global score, mean (SD)	63.4 (10.5)	68.3 (4.7)^a^	63.7 (7.5)^a^	45.3 (13.1)^a^	<0.001^d^
Classification					
Optimal	48 (40.7)	31 (63.3)^a^	17 (30.4)^a^	0 (0)^a^	<0.001^b^
Mildly reduced	18 (15.3)	10 (20.4)	8 (14.3)	0 (0)	NA
Moderately reduced	23 (19.5)	7 (14.3)	16 (28.6)	0 (0)	NA
Severely reduced	29 (24.5)	1 (2.0)^a^	15 (26.7)^a^	13 (100.0)^a^	<0.001^b^
ASQ‐TRAK, *n* = 120	120 (94.5)	51 (92.7)	58 (98.3)	11 (84.6)	NA
All on‐track domains	66 (55.0)	35 (68.6)^a^, ^c^	28 (48.3)^a^, ^c^	3 (27.3)^a^	0.015^b^
≥ 1 domain mild to moderate	24 (20.0)	9 (17.6) ^a,c^	14 (24.1) ^a,c^	1 (9.1)^a^	0.015^b^
≥ 1 domain severe	30 (0.25)	7 (13.7)^a^, ^c^	16 (27.6)^a^, ^c^	7 (63.6)^a^	0.003^b^

Abbreviations: ASQ‐TRAK, Ages and Stages Questionnaire‐Talking about Raising Aboriginal Kids; BM1, baby movement check 1; BM2, baby movement check 2; GMA, Prechtl's General Movements Assessment; HINE, Hammersmith Infant Neurological Examination; IQR, interquartile range; MOS‐R, Motor Optimality Score‐Revised; NDD, neurodevelopmental delay; 'on‐track'', typically developing.

Data are *n* (%) unless stated otherwise.

^a^ Significant difference (*p* < 0.05).

^b^ Fisher's exact test.

^c^ Non‐significant difference (*p* > 0.05).

^d^ Kruskal–Wallis with post‐hoc Dunn's.

The univariate and multivariate relationships between screening tools and neurodevelopmental outcomes are presented in Table 3, and Tables S2 and S3. The MOS‐R at 14 weeks or longer, HINE, and ASQ‐TRAK (≥1 domain, mild‐to‐severely reduced) distinguished infants developing ‘on track’ from NDD or CP at 12 months corrected age. The MOS‐R at 14 weeks or longer was the earliest biomarker (Multi_BM1), with the HINE as the strongest predictor at 4 to 9 months corrected age and overall (Multi_BM2, Multi_all). Each 1‐point decrease in HINE and MOS‐R increased the odds of NDD (OR_HINE_ = 1.12, 1.05–1.21; OR_MOS‐R_ = 1.40, 1.00–1.96) and CP (OR_HINE_ = 1.41, 1.21–1.65; OR_MOS‐R_ = 1.47, 1.08–2.01) compared to ‘on track’. Aberrant fidgety movements demonstrated a strong relationship with CP; however, it was not associated with high chance/confirmed NDD.

**TABLE 3:** Relationship between screening assessments and high‐chance or confirmed NDD or CP at 12 months (*n* = 127).

Screening tool	Model	High chance of NDD		High chance of CP	
		OR (95% CI)	*p*	OR (95% CI)	*p*
*Reference: 'on‐track' group*					
BM1 at 3–5 months					
GMA < 14 weeks					
Fidgety movements (absent)	Univariate	3.05 (0.32–28.70)	0.330	72.50 (5.49–958.01)	0.001
MOS‐R (lower)	Univariate	1.08 (0.95–1.26)	0.238	1.45 (1.18–1.78)	< 0.001
	Multi_BM1	0.94 (0.76–1.15)	0.055	1.33 (1.04–1.71)	0.025
	Multi_all	0.94 (0.75–1.18)	0.569	1.43 (1.00–2.06)	0.050
GMA ≥ 14 weeks					
Fidgety movements (absent)	Univariate	3.81 (0.39–infinity)	0.259	109.0 (13.82–infinity)	< 0.001
MOS‐R (lower)	Univariate	1.25 (1.04–1.49)	0.018	1.67 (1.33–2.10)	< 0.001
	Multi_BM1	1.40 (1.00–1.96)	0.047	1.47 (1.08–2.01)	0.014
	Multi_all	1.16 (0.84–1.62)	0.362	0.96 (0.63–1.46)	0.848
BM2 at 4–9 months					
HINE at 4–9 months					
Global score (lower)					
	Univariate	1.13 (1.05–1.22)	0.001	1.43 (1.28–1.67)	< 0.001
	Multi_BM2	1.12 (1.05–1.21)	0.002	1.41 (1.21–1.65)	0.002
	Multi_all	1.19 (1.03–1.37)	0.014	1.38 (1.05–1.81)	0.019
ASQ‐TRAK at 4–9 months					
≥ 1 domain mild to severe	Univariate	2.34 (1.07–5.13)	0.033	5.83 (1.36–24.94)	0.017
	Multi_BM2	1.77 (0.76–4.12)	0.187	0.94 (0.13–6.90)	0.956
	Multi_all	1.93 (0.52–7.22)	0.329	1.19 (0.03–56.48)	0.928
≥ 1 domain severe	Univariate	2.39 (0.90–6.40)	0.082	11.0 (2.54–47.59)	0.001
Trajectory					
MOS‐R < 23 + moderate to severely reduced^a^ HINE	Univariate	15.17 (4.16–55.37)	< 0.001	140.40 (18.49–infinity)	< 0.001

Abbreviations: ASQ‐TRAK, Ages and Stages Questionnaire‐Talking about Raising Aboriginal Kids; BM1, baby movement check 1; BM2, baby movement check 2; CI, confidence interval; CP, cerebral palsy; GMA, Prechtl's General Movements Assessment; HINE, Hammersmith Infant Neurological Examination; MOS‐R, Motor Optimality Score‐Revised; Multi_BM1, MOS‐R adjusted for screening assessments at both < 14 weeks and ≥ 14 weeks; Multi_BM2, adjusted for screening assessments at 4–9 months (HINE, ASQ‐TRAK); Multi_all, adjusted MOS‐R, HINE, and ASQ‐TRAK for all screening assessments; NDD, neurodevelopmental delay; 'on‐track', typically developing; OR, odds ratio.

^a^ Less than the significant delay cut‐off.

**FIGURE 1:**
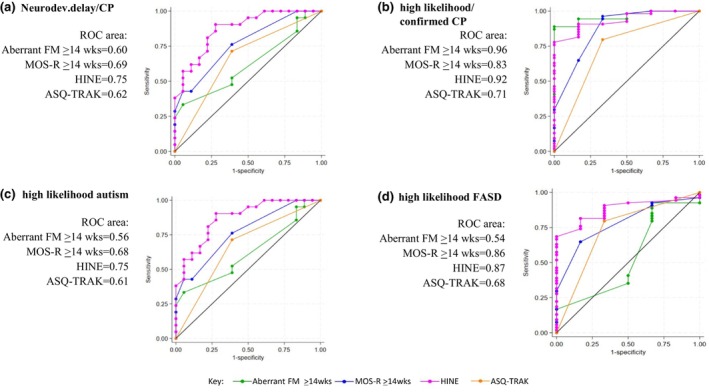
Receiver operating characteristic (ROC) curves for neurodevelopmental outcomes according to screening assessment. (a) Neurodevelopmental delay or CP. (b) High likelihood of or confirmed CP. (c) High likelihood of autism. (d) High likelihood of fetal alcohol spectrum disorder. Abbreviations: ASQ‐TRAK, Ages and Stages Questionnaire‐Talking about Raising Aboriginal Kids; CP, cerebral palsy; HINE, Hammersmith Infant Neurological Examination; MOS‐R, Motor Optimality Score‐Revised.

Receiver operator characteristic curve analyses were conducted to evaluate the diagnostic accuracy of early screening tools and determine the optimal cut‐off points for predicting neurodevelopmental outcomes. The cut‐off point was selected based on the Youden index, which identifies the threshold that maximizes the sum of sensitivity and specificity, thereby optimizing test performance.^39^ We opted to prioritize cut‐off scores with a higher sensitivity to identify more infants with a high chance of developmental concerns, rather than risk missing those who may benefit from early intervention or further diagnostic assessment. Analyses determined that an MOS‐R cut‐off of less than 23 at 14 weeks or longer best predicted high‐chance/confirmed NDD/CP (accuracy = 64%, sensitivity = 84%, specificity = 38%) (Figure 1 and Table S4). A moderately (less than the significant delay cut‐off) to severely (less than the CP cut‐off) reduced HINE score demonstrated the greatest diagnostic accuracy for NDD/CP (accuracy = 72%, sensitivity = 64%, specificity = 84%), with high PPV (85%). Aberrant fidgety movements were the strongest predictor of CP (accuracy = 94%, sensitivity = 73%, specificity = 97%).

A combined trajectory of MOS‐R (less than 23) and HINE (moderate‐to‐severely reduced) was a stronger predictor of high chance of NDD/CP than any single tool (Table 4 and Table S2), with 74% accuracy (sensitivity = 62%, specificity = 93%, PPV = 93%). For high chance of CP, the trajectories of the MOS‐R (less than 20) and HINE (severe) demonstrated similar accuracy (accuracy = 93%, sensitivity = 73%, specificity = 96%) to aberrant fidgety movements. Infants with CP were classified in GMFCS levels I and II (77%), which is predictive of independent ambulation, or in GMFCS levels III to V (23%), requiring greater functional support. A trajectory of MOS‐R (less than 9) and HINE (less than 40) was 100% accurate for severe CP (GMFCS levels IV and V), while a trajectory of asymmetry of finger posture (MOS‐R) and five or more asymmetries (HINE) was 92% accurate for unilateral CP (Table 4 and Table S5).

**TABLE 4:** Diagnostic accuracy of recommended screening tools at each time point for neurodevelopmental outcomes at 12 months.

Outcome	Recommended tool(s) and cut‐off score	Timing	Percentage accuracy (95% CI)	Percentage sensitivity (95% CI)	Percentage specificity (95% CI)
NDD or CP	MOS‐R (< 23)	14–16 weeks corrected age	64 (54–74)	84 (71–92)	38 (24–54)
	HINE (moderate to severe)^c^	4–9 months corrected age	72 (63–80)	64 (51–75)	84 (70–93)
	Trajectory: MOS‐R (< 23) + HINE (moderate to severe)		74 (65–82)	62 (49–74)	93 (81–99)
CP	Fidgety movements (aberrant)	14–16 weeks corrected age	94 (87–98)	73 (39–94)	97 (90–99)
	HINE (severe)^d^	4–9 months corrected age	86 (79–92)	100 (75–100)	85 (76–91)
	Trajectory: MOS‐R (< 20) + HINE (severely reduced)		93 (87–97)	73 (39–94)	96 (89–99)
Severity (GMFCS levels IV and V)	Trajectory: MOS‐R (<9) + HINE (<40)		100 (97–100)	100 (29–100)	100 (96–100)
Topography, unilateral CP	Trajectory: MOS‐R (asymmetry of finger postures) + HINE (≥ 5)^a^		92 (85–97)	60 (15–95)	94 (87–98)
Autism^b^	MOS‐R (< 23)	14–16 weeks corrected age	57 (43–69)	84 (64–95)	37 (22–55)
	HINE (mild to severe)^e^	4–9 months corrected age	68 (57–79)	74 (55–88)	64 (4–78)
	Trajectory: MOS‐R (< 23) + HINE (moderate to severe)		80 (68–89)	59 (39–78)	95 (82–99)
FASD^b^	MOS‐R (< 21)	14–16 weeks corrected age	73 (58–85)	83 (36–100)	71 (55–84)
	HINE (moderate to severe)	4–9 months corrected age	85 (73–93)	90 (52–100)	84 (70–93)
	Trajectory: MOS‐R (< 22) + HINE (moderate to severe)		92 (81–98)	89 (52–100)	93 (81–99)

Abbreviations: CI, confidence interval; CP, cerebral palsy; FASD, fetal alcohol spectrum disorder; GMFCS, Gross Motor Function Classification System; HINE, Hammersmith Infant Neurological Examination; MOS‐R, Motor Optimality Score‐Revised; NDD, neurodevelopmental delay.

^a^ Five or more asymmetries on the HINE.

^b^ Compared to developing on track.

^c^ Less than the significant delay cut‐off.

^d^ Less than the cerebral palsy cut‐off.

^e^ Less than the 10th centile.

### Prediction of CP, autism, and fetal alcohol spectrum disorder

A total of 47 of 127 (37%) infants were classified as confirmed or high chance of a specific NDD, including CP (*n* = 13), autism (*n* = 33), FASD (*n* = 9), and global developmental delay (*n* = 2). Ten infants (21%) had a co‐occurring high likelihood of autism.

Like NDD and CP, an MOS‐R of 14 weeks or longer and HINE differentiated infants with a high chance of FASD and autism from those developing ‘on track’, and were the strongest predictors of specific NDDs (Table S6). A combined trajectory of MOS‐R and HINE demonstrated the greatest overall diagnostic accuracy for FASD (sensitivity = 89%, specificity = 93%) and autism (sensitivity = 59%, specificity = 95%) (Table 4). Neurodevelopmental profiles significantly differed between infants with a high chance of CP, autism, and FASD (Table S7). Severe neurological delay (HINE, *p* = 0.006) and lower fine motor scores (PDMS‐2, *p* = 0.044) were associated with CP, while communication delay (Bayley‐III, *p* = 0.048) was associated with the likelihood of autism.

## DISCUSSION

In this prospective cohort study, we identified a strong relationship between early screening tools and 12‐month neurodevelopmental outcomes. Our findings support HINE as the strongest overall predictor of outcomes at 12 months corrected age, with a moderate‐to‐severely reduced score demonstrating the greatest accuracy for high‐chance/confirmed NDD, including autism and FASD or CP. At 3 to 5 months corrected age, the quality of an infant's movements and postures on the MOS‐R was a stronger early biomarker than fidgety movements alone.

Both the MOS‐R at 14 weeks or longer and HINE differentiated infants developing ‘on track’ from high chance of CP or other non‐CP NDD, including autism and FASD. An MOS‐R cut‐off score of less than 23 best predicted the overall neurodevelopmental outcome, correctly classifying 74% of infants, compared to 55% using fidgety movements alone. The MOS‐R demonstrated strong prognostic accuracy, with severely reduced scores (less than nine) predictive of severe CP (GMFCS levels III–V) and an asymmetry of finger postures on MOS‐R predictive of unilateral CP.

All infants with a high chance of CP demonstrated severely reduced HINE (less than the CP cut‐off score); when combined with an MOS‐R of less than 20, diagnostic accuracy improved compared to the HINE alone (93% vs 86%). Our findings demonstrate the transdiagnostic utility of the HINE as an early predictor of a high chance of autism and FASD, with reduced scores demonstrating strong predictive accuracy, suggesting that it may be one of the earliest clinical biomarkers for both conditions.^10^, ^15^

Early identification promotes timely referral to targeted interventions, optimizing individual developmental trajectories. Like previous studies,^16^, ^31^, ^32^, ^40^ we found a strong relationship between the HINE—a quantifiable, quick to administer (approximately 15 minutes), cost‐effective tool^29^—and neurodevelopmental outcomes. Infants who scored optimally were at lower risk for any NDD. The predictive ability of the HINE for the early identification of CP^13^, ^32^ has led to its broad real‐world implementation.^41^, ^42^, ^43^ Emerging evidence supports the predictive relationship between the HINE and cognitive,^16^, ^31^ motor,^40^ and mild neurological disabilities;^44^ however, to our knowledge, these findings are the first to report the predictive value of the HINE for a later autism or FASD diagnosis in any cohort.

Reflecting recommendations from the international clinical practice guidelines,^13^ and a well‐established body of evidence,^12^ absent fidgety movements remain the strongest predictor of CP. While no infants developing ‘on track’ demonstrated aberrant fidgety movements (i.e. no false positives), the presence of normal fidgety movements did not necessarily correlate with typical ‘on‐track’ development, reiterating the challenges of identifying the very early risk of non‐CP outcomes. While gestalt‐based fidgety movements may lack the sensitivity to identify mild‐to‐moderate delays,^17^, ^45^ the detailed GMA (MOS‐R)^27^ has strong utility for detecting non‐CP NDDs, with reported associations between lower MOS‐R and other NDDs.^14^, ^18^, ^19^, ^46^

Currently a MOS‐R cut‐off of less than 21 is recommended for referral to intervention services;^14^ however, published cut‐off scores for cognitive, motor, and developmental outcomes range from less than 21 to less than 24.^18^, ^47^, ^48^ Our broad cohort included all gestational ages (range = 23–41 weeks) with varied risk factors (very preterm birth, hypoxic‐ischaemic encephalopathy, low birthweight, neonatal surgery), with no significant difference in MOS‐R between infants born preterm and at term.^22^ This supports use of a universal MOS‐R cut‐off score across all risk exposures, as opposed to specific cut‐off scores based on gestational age.^25^, ^28^, ^49^, ^50^ Instead, we propose defining MOS‐R cut‐off scores for the likelihood of specific outcomes. In our cohort, MOS‐R cut‐off scores less than 23 best predicted mild‐to‐severe developmental concerns or high chance of NDDs, with a cut‐off‐score of less than 12 strongest for high‐chance/confirmed CP. We opted for cut‐off scores with higher sensitivity to ensure that we identified the greatest number of infants who would benefit from early intervention.

In the present study, no infants with CP scored more than 21 on the MOS‐R at 14 weeks or longer. Our study is the first to report predictive cut‐off scores for identifying a high chance of FASD (less than 21) and autism (less than 23). Both cut‐off scores demonstrated good sensitivity; however, specificity was low. While this was probably affected by the low MOS‐R scores of infants with other NDDs (motor/cognitive delay or CP), the transdiagnostic utility of the MOS‐R for identifying infants with clinical signs of autism and FASD is promising, promoting earlier and timely referral to therapeutic support than current practice (closer to school age).^51^

No single tool can predict the likelihood of a later outcome; rather, the trajectory of scores from the MOS‐R and HINE provide a snapshot of development in time. Previous studies reported the developmental trajectories of the GMA (writhing and fidgety movements) and HINE^52^, ^53^, ^54^ to predict neurodevelopmental outcomes. We add the predictive accuracy of a combined trajectory of MOS‐R and HINE. Higher MOS‐R and HINE scores were strongly associated with developing ‘on track’; conversely, a combined trajectory of low MOS‐R (less than 23) and moderate‐to‐severely reduced HINE demonstrated the greatest predictive accuracy for a high chance of NDD or CP at 12 months corrected age, improving specificity and PPV compared to either tool alone. While the ASQ‐TRAK was less predictive, results can improve our understanding of an infant's domain‐specific strengths and areas for support.^15^ More recently, Jackman et al.^18^ reported a predictive association between the MOS‐R and HINE at 3 months and infant neurodevelopment at 1 year; however, they found that combining scores did not improve the predictive value. Our findings support the necessity of triangulating results over a trajectory of multiple time points to gain a holistic understanding of an infant's overall clinical picture.^22^, ^52^

The LEAP‐CP programme represents a change from a ‘one‐size‐fits‐all’ health care approach to instead structure services based on the specific needs of the population. Rethinking and adapting neonatal follow‐up models of care requires cultural consultation and co‐design to embed First Nations peoples' priorities, world views, and ways of being, knowing, and doing into clinical service delivery. This process must also address structural and cultural barriers, recognize cultural facilitators, and incorporate infants' holistic risk profiles, including birth, maternal, cultural, and socio‐demographic factors, to guide individualized screening and support early and responsive care.

Strengths of our study are that it is the first to use an Australian First Nations culturally adapted early screening programme to investigate the transdiagnostic potential of evidence‐based tools to accurately predict infant developmental outcomes. The high retention rate of First Nations families, improving from 50% to 86%, compared to routine neonatal follow‐up, supports the importance of co‐designed programmes and culturally responsive health care. This study uniquely screened not only for a high chance of CP, but also other NDDs, to identify infants with high chance of autism and FASD earlier, in line with identified First Nations community priorities. Although rates of CP diagnosis among First Nations Australians are declining,^55^ the average age at diagnosis remains 19 months.^56^ In addition, First Nations children with CP often present with more complex gross motor profiles and may require greater functional and cognitive support.^55^, ^57^ In contrast, the reported prevalence of NDDs, including autism and FASD, in First Nations Australians remains variable. This variability is influenced by barriers to accessing specialist diagnostic services, limited awareness, and concerns around missed diagnosis or misdiagnosis,^58^, ^59^, ^60^ with diagnosis often delayed until school age. Our findings underscore the importance of early identification of all developmental concerns (including not only CP but also developmental delay and the potential for a later chance of autism and FASD) to enable timely referral to intervention services, irrespective of diagnostic certainty, thereby optimizing neurodevelopmental trajectories.

There are some potential limitations to our study. Outcomes at 12 months are accurate for the prediction of CP; however, the features for autism and FASD are less certain and require later follow‐up. Our study reported high rates of infants with an increased likelihood of autism.^58^, ^59^ The SACS‐R reported lower diagnostic accuracy for autism at 12 months compared to 18 months and 24 months,^36^ so our outcomes should be interpreted cautiously. Our study assessed four of the 10 developmental domains considered for FASD diagnosis,^38^ which were appropriate for the age of our cohort, with the remaining six domains only assessable in older children. Some confidence intervals for the odds ratios were notably wide, reflecting the imprecision of the estimate because of the small number of cases for specific NDDs.

### Conclusion

Our prospective cohort study of First Nations Australian infants provides the first evidence for the accuracy and transdiagnostic utility of detailed GMA (MOS‐R) and HINE in real‐world clinical settings to identify infants developing typically ‘on track’ and to predict a high chance of CP and other NDDs, including autism and FASD. These tools are feasible and scalable to neonatal follow‐up across broad health services in urban, regional, and remote settings, including First Nations communities and culturally diverse populations in low‐resource settings.

## Supporting information


**Figure S1:** Classification of screening and outcomes.

**Figure S2:** Study flow chart.

**Table S1:** Maternal characteristics and family demographics, *n* = 156.
**Table S2:** Relationship between screening assessments and high chance/confirmed NDD/CP at 12 months, *n* = 127.
**Table S3:** Relationship between HINE (categorical) and outcomes at 12 months, *n* = 127.
**Table S4:** Diagnostic accuracy of screening assessments (fidgety movements, MOS‐R, HINE, ASQ‐TRAK) to predict neurodevelopmental delay and/or cerebral palsy at 12 months corrected age.
**Table S5:** Relationship between early screening assessments (MOS‐R, HINE) and cerebral palsy severity and topography, *n* = 123.
**Table S6:** Relationship between screening assessments and high chance of CP, FASD and/or autism at 12 months, *n* = 127.
**Table S7:** Early screening outcomes and neurodevelopmental profile of infants with confirmed or high chance of CP, autism and/or FASD at 12 months.

## Data Availability

Data available on request from the authors: the data the support the findings of this study are available from the corresponding author upon reasonable request.
