# Supplementary material for: Predicting neurodevelopmental outcomes in Australian First Nations infants: The transdiagnostic utility of early screening tools
Source: Dev Med Child Neurol. 2025 Sep 25;68(3):381–93. doi: 10.1111/dmcn.70003 (PMC12875183; doi:10.1111/dmcn.70003)
Supplement: Supplementary file 1 — Figure S1: Classification of screening and outcomes. [file DMCN-68-381-s001.docx]

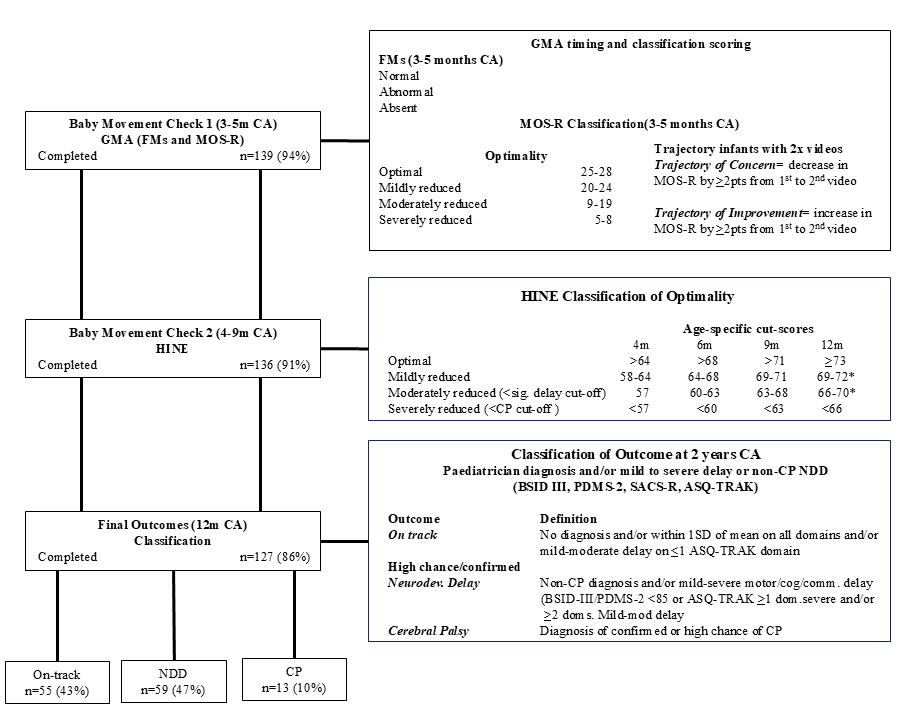


**Figure S1: Classification of screening and outcomes**

Abbreviations: ASQ-TRAK=Ages and Stages-Talking about Raising Aboriginal Kids, BSID-III=Bayley Scales of Infant and Toddler Development 3^rd^ Edition, CA=corrected age, CP=Cerebral Palsy, doms=domains, FMs=Fidgety movements, GMA=General Movements Assessment, HINE=Hammersmith Infant Neurological Examination, m=months, MOS-R=Motor Optimality Score-revised, n=number, NDD=Neurodevelopmental delay, PDMS-2=Peabody Developmental Motor Scales 2^nd^ edition, SACS-R=Social, Attention, Communication Surveillance-revised, SD=standard deviation, sig.delay=significant cognitive delay *12-month HINE classification was based on gestational age (term vs preterm).
