# Supplementary material for: Predicting neurodevelopmental outcomes in Australian First Nations infants: The transdiagnostic utility of early screening tools
Source: Dev Med Child Neurol. 2025 Sep 25;68(3):381–93. doi: 10.1111/dmcn.70003 (PMC12875183; doi:10.1111/dmcn.70003)
Supplement: Supplementary file 2 — Figure S2: Study flow chart. [file DMCN-68-381-s002.docx]

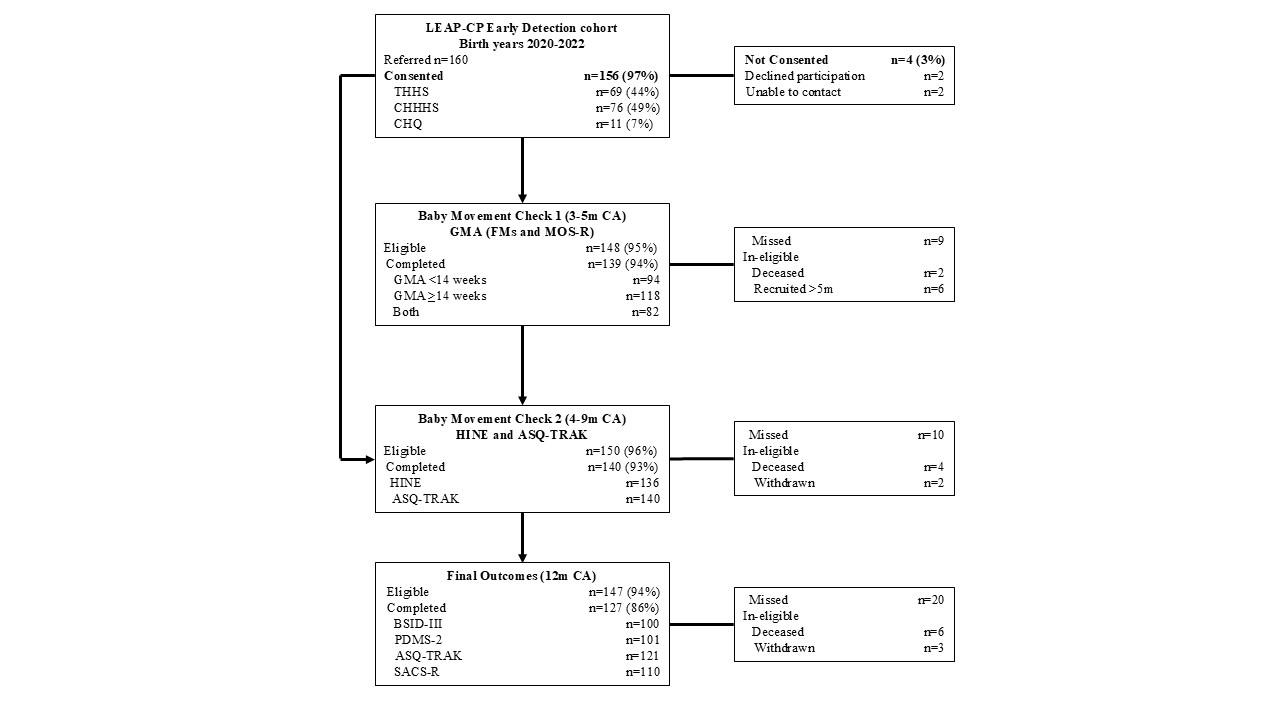
**Figure S2: Flow of study**

Abbreviations: ASQ-TRAK=Ages and Stages-Talking about Raising Aboriginal Kids, BSID-III=Bayley Scales of Infant and Toddler Development 3^rd^ Edition, CA=corrected age, CHQ=Children’s Health Queensland, CHHHS= Cairns and Hinterland Hospital and Health Service, FMs=Fidgety movements, GMA=General Movements Assessment, HINE=Hammersmith Infant Neurological Examination, LEAP-CP=Learning about Everyday Activities with Parents study, m=months, MOS-R=Motor Optimality Score-revised, n=number, THHS=Townsville Health and Hospital Service**.**
