# Supplementary material for: Predicting neurodevelopmental outcomes in Australian First Nations infants: The transdiagnostic utility of early screening tools
Source: Dev Med Child Neurol. 2025 Sep 25;68(3):381–93. doi: 10.1111/dmcn.70003 (PMC12875183; doi:10.1111/dmcn.70003)
Supplement: Supplementary file 3 — Table S1: Maternal characteristics and family demographics, n = 156. Table S2: Relationship between screening assessments and high chance/confirmed NDD/CP at 12 months, n = 127. Table S3: Relationship between HINE (categorical) and outcomes at 12 months, n = 127. Table S4: Diagnostic accuracy of screening assessments (fidgety movements, MOS‐R, HINE, ASQ‐TRAK) to predict neurodevelopmental delay and/or cerebral palsy at 12 months corrected age. Table S5: Relationship between early screening assessments (MOS‐R, HINE) and cerebral palsy severity and topography, n = 123. Table S6: Relationship between screening assessments and high chance of CP, FASD and/or autism at 12 months, n = 127. Table S7: Early screening outcomes and neurodevelopmental profile of infants with confirmed or high chance of CP, autism and/or FASD at 12 months. [file DMCN-68-381-s003.docx]

**Table S1: Maternal characteristics and family demographics, n=156**

|  | With 12m outcomes  Mean (SD) or n(%)  n=127 | W/out 12m outcomes  Mean (SD) or n(%)  n=29 | Missing/not completed n(%) |
| --- | --- | --- | --- |
| Maternal age ^+^ | 122 (96.1) | 26 (89.7) | 8 (11.5) |
| Age (years), mean (SD) | 27.6 (6.6) | 24 (5.7) |  |
| Age range | 16-43 | 16-36 |  |
| <20 | 15 (12.3) | 6 (23.1) |  |
| 20-25 | 30 (24.6) | 9 (34.6) |  |
| 25-35 | 57 (46.7) | 10 (38.5) |  |
| 35-40 | 16 (13.1) | 1 (3.8) |  |
| >40 | 4 (3.3) | 0 (0.0) |  |
| Maternal Education, n (%) | 86 (67.7) | 15 (51.7) | 55 (35.3) |
| University degree | 7 (8.1) | 0 (0.0) |  |
| TAFE/college certificate | 21 (24.4) | 2 (13.3) |  |
| Trade/apprenticeship | 3 (3.5) | 0 (0.0) |  |
| Year 12 | 14 (16.3) | 5 (33.3) |  |
| Less than year 12 | 41 (47.7) | 8 (53.3) |  |
| Child’s family care structure, n (%)^+^ | 125 (98.4) | 26 (89.7) | 5 (3.2) |
| One/both birth parents | 101 (80.8) | 26 (100.0) |  |
| Extended family/cultural adoption | 9 (7.2) | 0 (0.0) |  |
| Kinship care | 10 (8.0) | 0 (0.0) |  |
| Out of home care | 5 (4.0) | 0 (0.0) |  |
| Antenatal care | 92 (72.4) | 17 (58.6) | 47 (30.1) |
| Antenatal care (yes) | 83 (90.2) | 17 (100.0) |  |
| Limited or none | 9 (9.8) | 0 (0.) |  |
| Home language^+^ | 117 (92.1) | 18 (62.1) | 21 (13.5) |
| Cultural language and English | 30 (25.6) | 1 (5.6) |  |
| English only | 87 (74.4) | 17 (94.4) |  |

Abbreviations: n=number, TAFE=technical and further education, SD=standard deviation, w/out=without, %=percentage, ^+^=significant difference (p<0.05).

**Table S2: Relationship between screening assessments and high chance/confirmed NDD/CP at 12 months, n=127**

| Screening tool | Model | High chance NDD/CP | |
| --- | --- | --- | --- |
|  |  | OR (95% CI) | p-value |
| *Reference group: on track* | | | |
| *Baby Movement Check 1 at 3-5 months* | | | |
| GMA <14 wks | |  |  |
| FMs (absent) | univariable | 6.50 (0.78-54.4) | 0.08 |
| MOS-R (lower) | univariable | 1.11 (0.99-1.25) | 0.07 |
|  | Multi_BM1 | 1.01 (0.85-1.20) | 0.90 |
|  | Multi_all | 0.98 (0.80-1.20) | 0.86 |
| GMA>14 wks | |  |  |
| FMs(absent) | univariable | 1.42 (0.00-2.20) | 0.99 |
| MOS-R (lower) | univariable | 1.29 (1.10-1.53) | **0.002** |
|  | Multi_BM1 | 1.43 (1.07-1.91) | **0.015** |
|  | Multi_all | 1.14 (0.82-1.56) | 0.43 |
| *Baby movement Check 2 at 4-9 months* | | | |
| HINE 4-9 m | |  |  |
| Global score (lower) | univariable  Multi_BM2  Multi_all | 1.15 (1.07-1.23)  1.14 (1.06-1.22)  1.19 (1.04-1.36) | **<0.001**  **<0.001**  **0.012** |
| ASQ-TRAK 4-9 m |  |  |  |
| >1 dom.mild-sev | univariable | 2.68 (1.26-5.72) | **0.011** |
|  | Multi_BM2 | 1.71 (0.74-3.99) | 0.21 |
|  | Multi_all | 0.52 (0.14-1.92) | 0.33 |
| >1 dom.severe | univariable | 3.14 (1.23-8.06) | **0.017** |
| Trajectory |  |  |  |
| MOS-R <23 + mod.sev. HINE | univariable | 21.12 (5.87-75.95) | **<0.001** |

Abbreviations: ASQ-TRAK=Ages and Stages Talking about Raising Aboriginal Kids, CP=Cerebral Palsy, dom.=domain, FMs= fidgety movements, GMA=General movements assessment, HINE=Hammersmith Infant Neurological Examination, inf=infinity, m=months, mod.sev=moderate to severely reduced (<significant delay cut-off), MOS-R=Motor Optimality Score-revised, Multi_BM1=MOS-R adjusted for Screening assessments at both <14wks and >14wks, Multi_BM2=Adjusted for screening assessments at 4-9 months (HINE, ASQ-TRAK) Multi_all=adjusted MOS-R, HINE, ASQ-TRAK for all screening assessments, NDD=Neurodevelopmental delay, on track=typically developing, OR=odds ratio, wks=weeks, 95%CI=95% confidence intervals.

**Table S3: Relationship between HINE (categorical) and outcomes at 12 months, n=127**

| Screening tool | Multinomial logistic  Regression Model | High chance NDD | | High chance CP | |  |
| --- | --- | --- | --- | --- | --- | --- |
|  |  | OR (95% CI) | p-value | OR (95% CI) | p-value |  |
| *Reference group: on track* | | | | | |  |
| HINE 4-9 m | |  |  |  |  |  |
| mild-sev. reduced | univariable | 3.95 (1.75-8.91) | **0.001** | 29.06 (4.43-+inf) | **<0.001** |  |
| mod-sev. reduced | univariable | 6.36 (2.53-16.0) | **<0.001** | 80.14 (11.74- inf) | **<0.001** |  |
| sev. reduced | univariable | 17.55 (2.22-138.64). | **0.007** | 394.71 (47.21-+inf) | **<0.001** |  |
| >5 asymmetries | univariable | 2.11 (0.74-6.09) | 0.164 | 15.75 (3.67-67.52) | **<0.001** |  |
|  |  |  |  |  |  |  |
| Screening tool | Logistic  Regression Model | High chance NDD/CP | | | |  |
|  |  | OR (95% CI) | | p-value | |  |
| *Reference group: on track* | | | | | |  |
| HINE 4-9 m |  |  |  |  | |  |
| mild-sev. reduced | univariable | 5.27 (2.37-11.71) | | **<0.001** | |  |
| mod-sev. reduced | univariable | 9.02 (3.66-22.25) | | **<0.001** | |  |
| sev. reduced | univariable | 32.78 (4.27-251.53) | | **0.001** | |  |
| >5 asymmetries | univariable | 3.27 (1.21-8.85) | | **0.019** | |  |

Abbreviations: HINE=Hammersmith Infant Neurological Examination, m=months, mild-sev.=mild to severe (<10^th^ centile), mod-sev. reduced= moderate to severe (<significant delay cut-off), NDD=Neurodevelopmental delay, on track=typically developing, OR=odds ratio, sev. reduced=severely reduced (<CP cutoff), wks=weeks, 95%CI=95% confidence intervals.

| Screening tool | Confirmed/High chance of NDD and/or or Cerebral Palsy | | | | | |
| --- | --- | --- | --- | --- | --- | --- |
|  | Se % (95% CI) | Sp % (95% CI) | PPV % (95% CI) | NPV % (95% CI) | AUC (95% CI) | Correct class. % (95% CI) |
| GMA <14 wks, n=79 |  |  |  |  |  |  |
| FMs (aberrant) <14 wks | 18 (9-32) | 97 (83-100) | 90 (55-99) | 42 (38-46) | 0.57 (0.51-0.64) | 48 (37-60) |
| MOS-R (<23) <14 wks | 67 (52-80) | 43 (25-63) | 66 (57-74) | 45 (31-59) | 0.63 (0.48-0.78) | 58 (47-69) |
| GMA >14 wks, n=97 |  |  |  |  |  |  |
| FMs (aberrant) >14 wks | 20 (10-33) | 100 (92-100) | 100 (72-100) | 49 (46-52) | 0.60 (0.55-0.65) | 55 (44-65) |
| MOS-R (<23) >14 wks | 84 (71-92) | 38 (24-54) | 64 (58-70) | 64 (47-78) | 0.69 (0.57-0.83) | **64 (54-74)** |
| HINE, n=118 |  |  |  |  |  |  |
| Mild – severely reduced | 75 (64-85) | 63 (48-77) | 74 (66-81) | 65 (53-74) | 0.69 (0.61-0.78) | 70 (61-78) |
| Mod. – severely reduced | 64 (51-75) | 84 (70-93) | 85 (74-91) | 62 (54-70) | 0.75 (0.66-0.84) | **72 (63-80)** |
| Severely reduced | 41 (29-53) | 98 (89-100) | 97 (80-100) | 54 (49-59) | 0.69 (0.63-0.75) | 64 (55-73) |
| ASQ-TRAK, n= 120 |  |  |  |  |  |  |
| >1 dom. mild-severe | 55 (43-67) | 69 (54-81) | 70 (60-79) | 53 (45-61) | 0.62 (0.53-0.71) | 61 (52-70) |
| >1 dom. severe | 33 (22-46) | 86 (74-94) | 77 (60-88) | 49 (44-54) | 0.60 (0.52-0.67) | 56 (46-65) |
| MOS-R (<23) + HINE (mod-sev) | 62 (49-74) | 93 (81-99) | 93 (81-98) | 62 (54-69) | 0.77 (0.70-0.84) | **74 (65-82)** |
| Screening tool | Confirmed/High chance of Cerebral Palsy | | | | | |
|  | Se % (95% CI) | Sp % (95% CI) | PPV % (95% CI) | NPV % (95% CI) | AUC (95% CI) | Correct class. % (95% CI) |
| GMA <14 wks, n=79 |  |  |  |  |  |  |
| FMs (aberrant) | 71 (29-96) | 93 (85-98) | 50 (28-72) | 97 (91-99) | 0.82 (0.64-1.00) | 91 (83-96) |
| MOS-R (<20) | 86 (42-100) | 89 (79-95) | 43 (27-61) | 98 (91-100) | 0.87 (0.73-1.00) | 89 (80-95) |
| GMA >14 wks, n=97 |  |  |  |  |  |  |
| FMs (aberrant) | 73 (39-94) | 97 (90-99) | 73 (45-90) | 97 (91-99) | 0.96 (0.85-0.99) | **94 (87-98)** |
| MOS-R (<20) | 73 (39-94) | 93 (86-97) | 57 (36-76) | 96 (91-99) | 0.83 (0.69-0.97) | 91 (83-96) |
| HINE, n=118 |  |  |  |  |  |  |
| Mild – severely reduced | 100 (75-100) | 46 (36-56) | 19 (16-21) | 100 (93-100) | 0.73 (0.68-0.78) | 52 (42-61) |
| Mod. - severely reduced | 100 (75-100) | 63 (53-72) | 25 (21-30) | 100 (95-100) | 0.81 (0.77-0.86) | 67 (58-75) |
| Severely reduced | 100 (75-100) | 85 (76-91) | 45 (34-56) | 100 (96-100) | 0.92 (0.89-0.96) | **86 (79-92)** |
| ASQ-TRAK, n= 120 |  |  |  |  |  |  |
| >1 dom. mild-severe | 73 (39-94) | 58 (48-67) | 15 (10-21) | 95 (89-98) | 0.65 (0.51-0.79) | 59 (50-68) |
| >1 dom. severe | 64 (31-89) | 79 (70-86) | 23 (15-35) | 96 (91-98) | 0.71 (0.56-0.87) | 78 (69-85) |
| MOS-R (<20) + HINE (sev) | 73 (39-94) | 96 (89-99) | 67 (42-85) | 97 (92-99) | 0.84 (0.70-0.98) | **93 (87-97)** |

**Table S4: Diagnostic accuracy of screening assessments (FMs, MOS-R, HINE, ASQ-TRAK) to predict Neurodevelopmental delay and/or cerebral palsy at 12 months corrected age**

Abbreviations: ASQ-TRAK=Ages and Stages Questionnaire-Talking about Raising Aboriginal Kids, AUC=Area under the ROC Curve, Correct class=correctly classified, FMs=Fidgety Movements, GMA=General Movements Assessment, HINE=Hammersmith Infant Neurological Examination, MOS-R=Motor Optimality Score-revised, n=number, NPV=negative predictive value, PPV=positive predictive value, Se=sensitivity, sev-severe, Sp=specificity, wks=weeks, 95%CI=95% confidence interval.

**Table S5: Relationship between early screening assessments (MOS-R, HINE) and cerebral palsy severity and topography, n=123**

|  | CP severity – GMFCS IV-V | | | | |
| --- | --- | --- | --- | --- | --- |
|  | OR (95% CI) | p-value | Se (95% CI) | Sp (95% CI) | Acc. (95% CI) |
| MOS-R sev. red (<9) |  |  |  |  |  |
| MOS-R<14wks | 173.33 (11.0-+inf) | **<0.001** | 100 (16-100) | 100 (95-100) | 100 (95-100) |
| MOS-R>14wks | 168.18 (14.95-+inf) | **<0.001** | 100 (29-100) | 99 (94-100) | 99 (94-100) |
| HINE |  |  |  |  |  |
| HINE <40 | 396.20 (31.36-+inf) | **<0.001** | 100 (29-100) | 100 (97-100) | 100 (97-100) |
| Trajectory  MOS-R (<9) + HINE (<40) | 351.37 (27.80-+inf) | **<0.001** | 100 (29-100) | 100 (96-100) | 100 (97-100) |
| CP topography – Unilateral CP | | | | | |
|  | OR (95% CI) | p-value | Se (95%CI) | Sp (95%CI) | Acc. (95%CI) |
| MOS-R (asym.finger) |  |  |  |  |  |
| MOS-R<14wks | 2.20 (0.21-23.27) | 0.512 | 25 (1-81) | 87 (77-94) | 84 (74-91) |
| MOS-R>14wks | 7.70 (1.18-50.09) | **0.033** | 60 (15-95) | 84 (75-91) | 82 (73-89) |
| HINE |  |  |  |  |  |
| asymmetry (>5) | 19.13 (2.13-171.89) | **0.008** | 83 (36-100) | 79 (71-86) | 79 (71-86) |
| Trajectory  MOS-R (asym.fing) + HINE (>5) | 23.25 (3.24-166.80) | **0.002** | 60 (15-95) | 94 (87-98) | 92 (85-97) |

Abbreviations: Acc.=accuracy, asym. finger=asymmetry of finger postures on MOS-R, CP=Cerebral Palsy, GMFCS=Gross Motor Function Classification System, HINE=Hammersmith Infant Neurological Examination, MOS-R=Motor Optimality Score-revised, OR=odds ratio, Se=sensitivity, Sp=specificity, sev. red=severely reduced, wks=weeks, 95% CI=95% confidence interval

| Screening tool | Model | Neurodevelopmental Outcome | | | | | | |
| --- | --- | --- | --- | --- | --- | --- | --- | --- |
|  |  | High chance of Cerebral Palsy | | High chance of FASD | | High chance of autism | |  |
|  |  | OR (95%CI) | p | OR (95%CI) | p | OR (95%CI) | p | R2 |
| *Reference group: on-track* |  |  |  |  |  |  |  |  |
| *Baby Movement Check 1 at 3-5 months* | | | | | | | |  |
| GMA <14wks  FMs (absent) | univariable | 72.5 (5.5-958.01) | **0.001** | 8.29 (0.65-104.89 | 0.103 | 4.35 (0.42-44.88) | 0.217 | 0.10 |
| MOS-R (lower score) | univariable | 1.35 (1.12-1.63) | **0.002** | 1.12 (0.95-1.32) | 0.198 | 1.10 (0.96-1.26) | 0.161 | 0.10 |
|  | Multi_BM1 | 1.36 (1.03-1.79) | **0.03** | 0.84 (0.54-1.32) | 0.45 | 0.89 (0.67-1.20) | 0.45 | 0.25 |
|  | Multi_all | 1.42 (0.95-2.13) | 0.09 | 0.56 (0.26-1.30) | 0.19 | 0.81 (0.55-1.21) | 0.30 | 0.41 |
| GMA >14wks  FMs (absent) | univariable | 109.0 (13.82-+inf.) | **<0.001** | 19.71 (1.45-+inf) | 0.03 | 5.77 (0.60-+inf) | 0.13 | 0.17 |
| MOS-R (lower score) | univariable | 1.71 (1.34-2.17) | **<0.001** | 1.47 (1.13-1.91) | **0.005** | 1.34 (1.06-1.69) | **0.014** | 0.19 |
|  | Multi_BM1 | 1.93 (1.03-1.79) | **0.02** | 2.74 (1.33-5.66) | **0.006** | 2.44 (1.31-4.55) | **0.005** | 0.25 |
|  | Multi_all | 1.25 (0.61-2.56) | 0.55 | 2.53 (0.86-7.47) | 0.09 | 1.61 (0.77-3.36) | 0.21 | 0.41 |
| *Baby movement Check 2 at 4-9 months* | | | |  |  |  |  |  |
| HINE 4-9 m |  |  |  |  |  |  |  |  |
| Global (lower score) | univariable | 1.24 (1.08-1.42 | **0.002** | 1.21 (1.10-1.33) | **<0.001** | 1.18 (1.09-1.28) | **<0.001** | 0.20 |
|  | Multi_BM2 | 1.27 (1.14-1.41) | **<0.001** | 1.19 (1.08-1.32) | **0.001** | 1.16 (1.07-1.26) | **<0.001** | 0.20 |
|  | Multi_all | 1.33 (1.01-1.75) | **0.04** | 1.35 (1.03-1.77) | **0.03** | 1.40 (1.09-1.79) | **0.009** | 0.41 |
| Mild-sev. reduced | univariable | 29.05 (4.43-+inf) | **<0.001** | 13.78 (1.59-119.29) | **0.017** | 4.95 (1.83-13.35) | **0.002** | 0.13 |
| Mod-sev. reduce | univariable | 80.14 (11.74-+inf) | **<0.001** | 41.0 (4.49-374.75) | **0.001** | 6.22 (2.21-17.55) | **0.001** | 0.20 |
| Severely reduced | univariable | 394.71 (47.2-+inf) | **<0.001** | 96.01 (8.56-1076.6) | **<0.001** | 22.86 (2.75-190.17) | **0.004** | 0.26 |
| ASQ-TRAK 4-9 m |  |  |  |  |  |  |  |  |
| >1 dom.mild-sev | univariable | 5.83 (1.36-24.94) | **<0.001** | 4.34 (0.97-19.74) | 0.06 | 2.33 (0.93-5.85) | 0.07 | 0.04 |
| >1 dom.severe | univariable | 11.0 (2.54-47.59) | **0.001** | 5.03 (1.08-23.40) | **0.04** | 3.46 (1.17-10.23 | **0.03** | 0.06 |
|  | Multi_BM2 | 1.31 (0.16-10.89) | 0.80 | 1.98 (0.322-12.15) | 0.46 | 1.90 (0.55-6.60) | 0.31 | 0.20 |
|  | Multi_all | 5.42 (0.10-282.48) | 0.40 | 5.97 (0.19-188.94) | 0.31 | 1.26 (0.07-21.81) | 0.87 | 0.41 |
| Trajectory |  |  |  |  |  |  |  |  |
| MOS-R <23 + mod-sev. HINE | univariable | 140.40 (18.49-+inf) | **<0.001** | 104.0(9.56-1132.5) | **<0.001** | 18.91 (4.65-76.91) | **<0.001** | 0.27 |

**Table S6: Relationship between screening assessments and high chance of CP, FASD and/or autism at 12 months, n=127**

Abbreviations: ASQ-TRAK=Ages and Stages-Talking about Raising Aboriginal Kids, CP=cerebral palsy, dom.=domain, Developmental delay=infants with mild-severe motor, cognition or communication delay, FM=fidgety movements, GMA=General Movements Assessment, HINE=Hammersmith Infant Neurological Examination, mod-sev.=moderate to severe, MOS-R=Motor Optimality Score-revised, Multi_BM1=MOS-R adjusted for Screening assessments at both <14wks and >14wks, Multi_BM2=Adjusted for screening assessments at 4-9 months (HINE, ASQ-TRAK) Multi_all=adjusted MOS-R, HINE, ASQ-TRAK for all screening assessments, on-track=typically developing, OR=odds ratio, p=p-value, wks=weeks, 95%CI=95% confidence interval.

**Table S7: Early screening outcomes and neurodevelopmental profile of infants with confirmed or high chance of CP, autism and/or FASD at 12 months**

| Developmental  domain | Confirmed or high chance | | |  |
| --- | --- | --- | --- | --- |
|  | Cerebral Palsy  n=13 | Autism  n=33 | FASD  n= 9 | p-value |
| Gross motor, n (%) | 11 (77.0) | 33 (100.0) | 9 (100.0) |  |
| PDMS-2, mean (SD) | 69.1 (19.7) | 84.0 (16.9) | 82.8 (17.8) | 0.084^**^ |
| Med (IQR) | 67 (53.0-91.0) | 90.0 (74.0-96.0) | 88 (72.0-93.5) |  |
| Delay yes, n(%) | 7 (63.6) | 12 (36.4) | 4 (44.4) | 0.283^*^ |
| Mild-mod | 0 (0.0) | 4 (33.3) | 0 (0.0) |  |
| Severe | 7 (100.0) | 8 (66.7) | 4 (100.0) | 0.052^*^ |
| Fine motor, n(%) | 11 (77.0) | 33 (100.0) | 9 (100.0) |  |
| PDMS-2, mean (SD) | 68.0 (14.2) ^^+^ | 82.7 (16.6) ^^^ | 84.3 (17.7) ^^+^ | **0.044**^**^ |
| Med (IQR) | 73 (64.0-79.0) | 82.0 (76.0-94.0) | 82 (70.0-98.5) |  |
| Delay yes, n(%) | 11 (100.0) | 22 (66.7) | 7 (77.8) | 0.072^*^ |
| Mild-mod | 5 (45.5) | 12 (54.5) | 4 (57.1) |  |
| Severe | 6 (54.5) | 10 (45.5) | 3 (42.9) | 0.343^*^ |
| Cognition, n(%) | 10 (76.9) | 32 (97.0) | 9 (100.0) |  |
| BSID-III, mean (SD) | 68.5 (16.2) | 76.9 (16.3) | 76.4 (16.0) | 0.354^**^ |
| Med (IQR) | 62.5 (55.0-80.0) | 75.0 (60.0-90.0) | 75.0 (60.0-95.0) |  |
| Delay yes, n(%) | 8 (80.0) | 24 (75.0) | 7 (77.8) | 0.944^*^ |
| Mild-mod | 1 (12.5) | 10 (41.7) | 4 (57.1) |  |
| Severe | 7 (87.5) | 14 (58.3) | 3 (42.9) | 0.181^*^ |
| Communication, n(%) | 11 (84.6) | 32 (97.0) | 9 (100.0) |  |
| BSID-III, mean (SD) | 71.4 (18.5) | 66.1 (13.1) | 73.7 (22.8) | 0.706^**^ |
| Med (IQR) | 68.0 (56.0-86.0) | 68.0 (56.0-71.0) | 71.0 (56.0-94.0) |  |
| Delay yes, n(%) | 7 (63.6) ^^+^ | 30 (93.8) ^+^ | 7 (77.8) ^^+^ | **0.048**^*^ |
| Mild-mod | 2 (28.6) | 10 (33.3) | 2 (28.6) |  |
| Severe | 5 (71.4) | 20 (66.7) | 5 (71.4) | 0.951^*^ |
| Neurological, n(%) | 11 (84.6) | 31 (93.9) | 6 (66.7) |  |
| HINE, mean (SD) | 49.2 (19.7)^^+^ | 63.8 (13.7) ^^+^ | 62.6 (6.1) ^^^ | **0.031**^**^ |
| Med (IQR) | 52.5 (25.5-65.5) | 68.0 (61.5-70.0) | 61.5 (59.0-69.5) |  |
| Delay yes, n(%) | 11 (100.0) | 28 (90.3) | 6 (100.0) | 0.403^*^ |
| Mild | 1 (9.1) | 7 (25.0) | 1 (16.7) |  |
| Moderate | 1 (9.1) | 12 (42.9) | 1 (16.7) |  |
| Severe | 9 (81.8) ^^+^ | 9 (32.1) ^+^ | 4 (66.6) ^^+^ | **0.006**^*^ |
| Eligible for neonatal f/up, | 11 (84.6) | 22 (66.7) | 8 (88.9) | 0.313 |

Abbreviations: BSID-III=Bayley Scales of Infant Development 3^rd^ edition, FASD=Fetal Alcohol Spectrum Disorder, f/up=follow up, HINE=Hammersmith Infant Neurological Examination, IQR=interquartile range, Med=median, mild-mod.=mild to moderate, n=number, PDMS-2=Peabody Developmental Motor Scales 2^nd^ edition, SD=standard deviation, ^*^=Fisher’s exact, ^**^=Kruskal-Wallis test.

10 infants had co-occuring high likelihood of autism and CP or FASD.

# Supporting Information

**Figure S1: Classification of screening and outcomes**


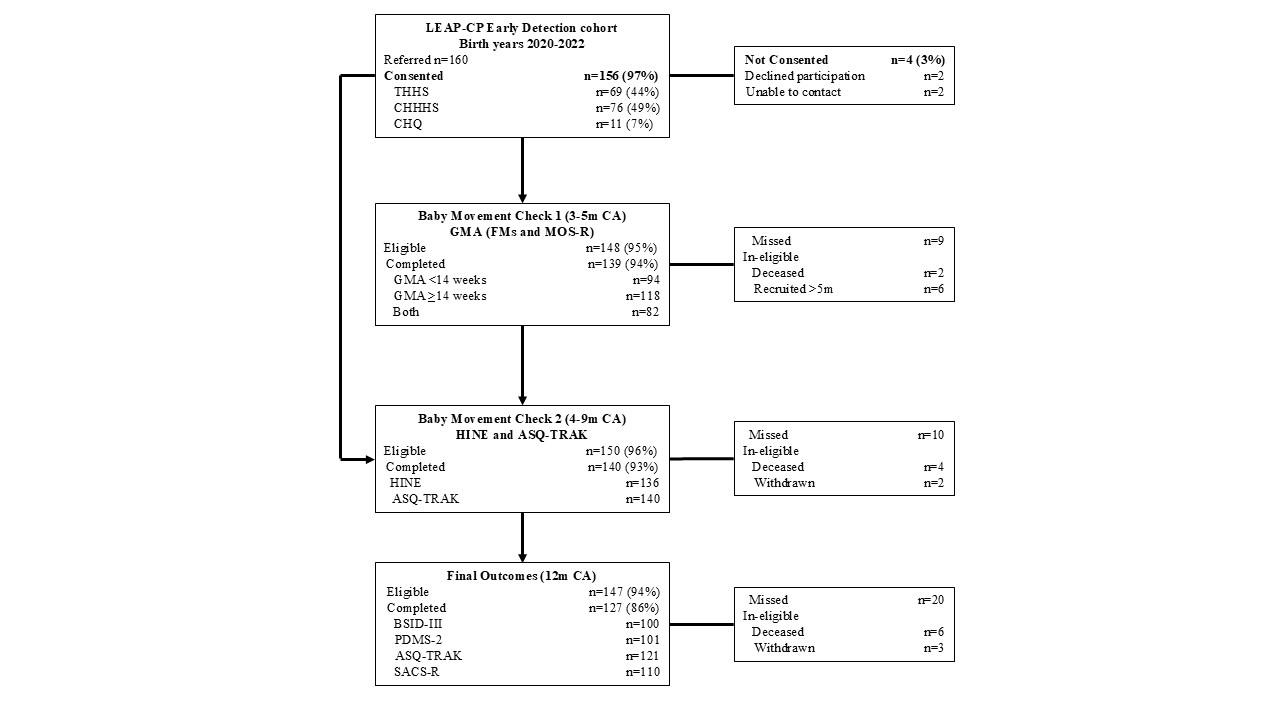
**Figure S2: Flow of study**
